# Resistance to protease inhibitors among persons living with HIV in Ghana: a case for viral load and drug resistance monitoring

**DOI:** 10.1186/s12985-024-02354-6

**Published:** 2024-07-20

**Authors:** Makafui Seshie, Billal Musah Obeng, Vivian Etsiapa Boamah, Marcel Bayor, Evelyn Yayra Bonney, Stephen Yao Gbedema, Kwamena William Coleman Sagoe

**Affiliations:** 1 Department of Medical Microbiology, School of Biomedical and Allied Health Sciences, University of Ghana, Accra, Ghana; 2 Department of Pharmaceutics, College of Health Sciences, Kwame Nkrumah University of Science and Technology, Kumasi, Ghana; 3 Immunovirology and Pathogenesis Program, Kirby Institute, University of New South Wales, Sydney, Australia; 4 Department of Virology, Noguchi Memorial Institute for Medical Research, University of Ghana, Accra, Ghana

**Keywords:** Second-line ART, Protease inhibitors, Treatment failure, HIV-1 viral load, HIV-1 drug resistance test, HIV care continuum

## Abstract

**Objective:**

Routine viral load and drug resistance testing are well supported in most resource-rich settings and provide valuable benefits in the clinical care of PLWH in these communities. Undoubtedly, there exist financial and political constraints for the scale-up of viral load and drug resistance testing in Sub-Saharan Africa. To achieve the global UNAIDS 95/95/95 targets, there is the need to bridge this inequity in patient care and allow for a universal approach that leaves no community behind.

**Methods:**

Venous blood from 96 PLWH on second-line ART from Korle-Bu Teaching Hospital were collected and processed into plasma for CD4^+^ T- cell and viral load assessments. Ribonucleic acid (RNA) was extracted from stored plasma and the protease gene amplified, sequenced and analyzed for subtype and drug resistance mutations using the Stanford HIV drug resistance database.

**Results:**

Out of the 96 PLWH, 37 experienced virological failure with 8 patients’ samples successfully sequenced. The predominant HIV-1 subtype identified was CRF02_AG (6/8, 75.0%) with 12.5% (1/8) each of CFR06_cpx infection and one case unable to subtype. The major PI resistance mutations identified were; M46I, I54V, V82A, I47V, I84V and L90M.

**Conclusions:**

Persons living with HIV who had experienced virologic failure in this study harboured drug resistance mutations to PI, thus compromise the effectiveness of the drugs in the second line. Resistance testing is strongly recommended prior to switching to a new regimen. This will help to inform the choice of drug and to achieve optimum therapeutic outcome among PLWH in Ghana.

## Highlights

Protease inhibitors (PI) are prescribed for patients failing on reverse transcriptase-based regimen without adequate monitoring for viral load and drug resistance testing.

The study provides evidence of PI resistance mutations and their implications for the relatively fewer individuals on PI-based regimen in the era of dolutegravir (DTG) use.

Close monitoring on all HIV infected patients on PI is needed.

## Introduction

At the end of 2022, an estimated number of 39 million people were living with HIV (PLWH) globally with 25.6 million in Africa [1]. Within the same year, 1.3 million new infections and 630,000 deaths from AIDS-related illnesses were reported [1]. In Ghana, 354,927 people were living with HIV with prevalence rate of 1.7% at the end of 2021 [2, 3]. Since the scale up of anti-retroviral therapy (ART), gradual increase in resistance to the drugs in Sub-Saharan Africa has been reported in several studies which has warranted current recommendations on ARTs for treatment and prevention [4, 5]. Protease inhibitors (PI) generally have high genetic barrier to the selection of ART resistant variants, which means after failure with first line drugs, suppression with PI-based regimen could be achieved [6]. Even though there is increased ART coverage, the price of PI such as ritonavir-boosted lopinavir (LPV/r) and ritonavir-boosted atazanavir (ATV/r) are still about two-fold higher than the first-line drugs in low resource countries [7].

Genotyping to confirm drug resistance mutations is key to inform therapy especially in cases where viremia is detected [8, 9]. However, this is not the case for most resource-limited settings including Ghana where genotypic antiretroviral testing (GART) presents financial and technical burden. Present data from the Ghana AIDS Commission (GAC) on the UNAIDS 95-95-95 target reveals a deficiency in optimizing GART to guide therapy and achieve the UNAIDS targets [2]. Better therapeutic outcome from second-line regimen amongst persons failing first-line therapy in Ghana is of great concern due to the extensive use of PI as the ART of choice in second and third-line regimens [10]. Even though, recently, there are integrase inhibitors such as dolutegravir (DTG) rolled-out in the ART guidelines in Ghana and other low-and middle-income countries [10–12], their cost and accessibility has been factors for their relatively slower use in patients’ regimen [13]. Currently, there is still a minority of patients still on PI, but with emphasis on DTG therapy it will be important to understand the dynamics of PI resistance to enable proper monitoring of PLWH on PIs. This study therefore sought to determine drug resistance mutations to PI containing regimen among PLWH virologically failing second-line antiretroviral therapy in Ghana.

## Methods

### Study design and population

A total of 96 PLWH on antiretroviral regimen consisting of two nucleoside reverse transcriptase inhibitors (NRTIs) and one PI consented and enrolled onto the study between July 2016 and June 2017.

### Laboratory analysis

The CD4^+^ T-cell count and plasma HIV-1 viral load were determined at the Fever’s unit laboratory immediately after sample collection. All viral RNA extraction, complementary DNA synthesis, nested-polymerase chain reaction and sequencing were done as previously published [14].

### Phylogenetic analysis

Nucleotide sequences for each sample were assembled to form a contig using SeqManPro 13 (DNASTAR Incorporation, U.S.A). Consensus sequence obtained was aligned with an HIV reference sequence (B-HXB2-PRT_2253–3700) in BioEdit (http://www.mbio.ncsu.edu/Bioedit/bioedit.html). Sequences were submitted to the Stanford University HIV Drug Resistance Database (https://hivdb.stanford.edu/hivdb/by-sequences/) to assign subtypes and analysed for HIV drug resistance mutations. The subtypes were confirmed with the Los Alamos National Laboratory HIV Database (http://www.hiv.lanl.gov). Neighbor-joining tree with the Kimura’s 2-parameter distances was employed in Molecular Evolutionary Genetic Analysis version 6.0 (MEGA 6).

### Statistical analysis

The mean CD4 count and mean viral load were calculated across patient demographics and statistical significance determined at 95% confidence level via non-parametric tests- Mann-Whitney U test for two patient categories (sex, adherence at 1 week and adherence at 1 month) and Kruskal-Wallis H test for four patient categories (age group & education). All analyses were done with Microsoft Excel and Statistical Package for Social Science (SPSS version 22).

## Results

### Study population

Ninety-six (96) PLWH were enrolled in this study. Most of the respondents 66% (*n* = 63) were above 40 years and were females, 70% (*n* = 67). A high non-adherence level of 96% (*n* = 92) was observed in the week of medication, which increased to 97% (*n* = 93) after a month where one patient was reported to be non-adherent after initial adherence at 1 week (Table 1). Importantly, there was significant reduction in viral load copies within 1 month of adherence but not with CD4^+^ T cell count. Also, significance was observed for viral load copies with educational level where patients with tertiary level education had the lowest relative mean viral load count of 4542.09 copies/ml and those without any formal education reported to have the highest mean viral load of 143697.40 copies/ml (Table 1).

**Table 1:** Demographic characteristics of patients

Variable	Frequency *n* = 96 (%)	CD4 + count (cells/ul)		Mean viral load (copies/ml)	
		Mean	p-value	Mean	p-value
**Age categories (years)**					
< 30	4 (4)	244.17		39327.93	
30–40	29 (30)	455.36	0.190	45448.91	0.354
> 40	63 (66)	419.16		28708.40	
**Sex**					
Male	29 (30)	356.41	0.088	30601.29	0.074
Female	67 (70)	451.54		35768.98	
**Educational Level**					
Basic	15 (16)	376.09		49421.15	
Secondary	51 (53)	430.30	0.608	19898.55	0.023*
Tertiary	20 (21)	466.57		4542.09	
N/A	10 (10)	367.13		143697.40	
**Adherence (7 days)**					
No	92 (96)	423.87	0.840	34676.15	0.864
Yes	4 (4)	398.25		23438.25	
**Adherence (30 days)**					
No	93 (97)	420.66	0.704	35618.83	0.001*
Yes	3 (3)	461.93		8529.14	

*Significance at 95% confidence level

**Table 2:** Predicted susceptibility and drug resistance profiles of study participants

ID	AGE	CD4^+^	VL	ART_D_/ yrs	ART Regimen	Subtype	PI Mutations	Drug Susceptibilities							
								LPV/r	ATV/r	NFV/r	DRV/r	FRV/r	IDV/r	SQV/r	TPV/r
PI007	47	27	2508	6	ATV/r 3TC/ZDV	CRF02-AG	M46I, Q58E, L76V, 184 V	H	H	S	I	S	S	S	S
PI009	18	284	76,808	4	LPV/r 3TC/ZDV	CRF02-AG	None	S	S	S	S	S	S	S	S
PI011	49	97	48,497	6	ATV/r 3TC/TDF	None	None								
PI015	70	5	217,264	9	LPV/r 3TC/TDF	CRF02-AG	K43KT, M46MI, I54IV, L76LV, V82VA	H	H	H	L	S	S	S	S
PI019	44	64	220,144	3	LPV/r 3TC/TDF	CRF06-CPX	M46I, I47V, I54V, V82A, L90M, L23I, F53L	H	H	S	P	S	S	S	S
PI039	48	60	44,614	3	LPV/r 3TC/ZDV	CRF02-AG	None	S	S	S	S	S	S	S	S
PI040	29	197	43,164	8	LPV/r TDF/FTC	CRF02-AG	None	S	S	S	S	S	S	S	S
PI069	35	12	154,490	7	LPV/r TDF/FTC	CRF02-AG	None	S	S	S	S	S	S	S	S

**Key**: HIV DRM_PI_: HIV Drug Resistance Mutation in protease gene, D: duration, NA: not available. **CRFO2-AG**; circulating recombinant form, **CRO6-CPX;** circulating recombinant form 06-cpx, **S**; Susceptible, **L**; Low resistance, **I** -Intermediate resistance, **P** -Potential low resistance, **H**; High resistance

### ART prescriptions for enrolled patients

Records from patient folders indicated that they were on second-line ART regimen which were primarily made of two of NRTIs; Lamivudine (3TC), Emtricitabine (FTC), Tenofovir (TDF), Zidovudine (ZDV), and either LPV/r or ATV/r as PI in the following combinations; TDF/FTC/LPV/r- 10 (9.8%), 3TC/TDF/LPV/r- 43 (42.2%), 3TC/TDF/ATV/r- 15 (14.7%), 3TC/ZDV/LPV/r- 20 (19.6%), 3TC/ZDV/ATV/r- 13 (12.7%) and TDF/FTC/ATV/r- 1 (0.9%) (Table 2).

### Phylogenetic analysis of *PR* gene sequences

**Fig. 1:**
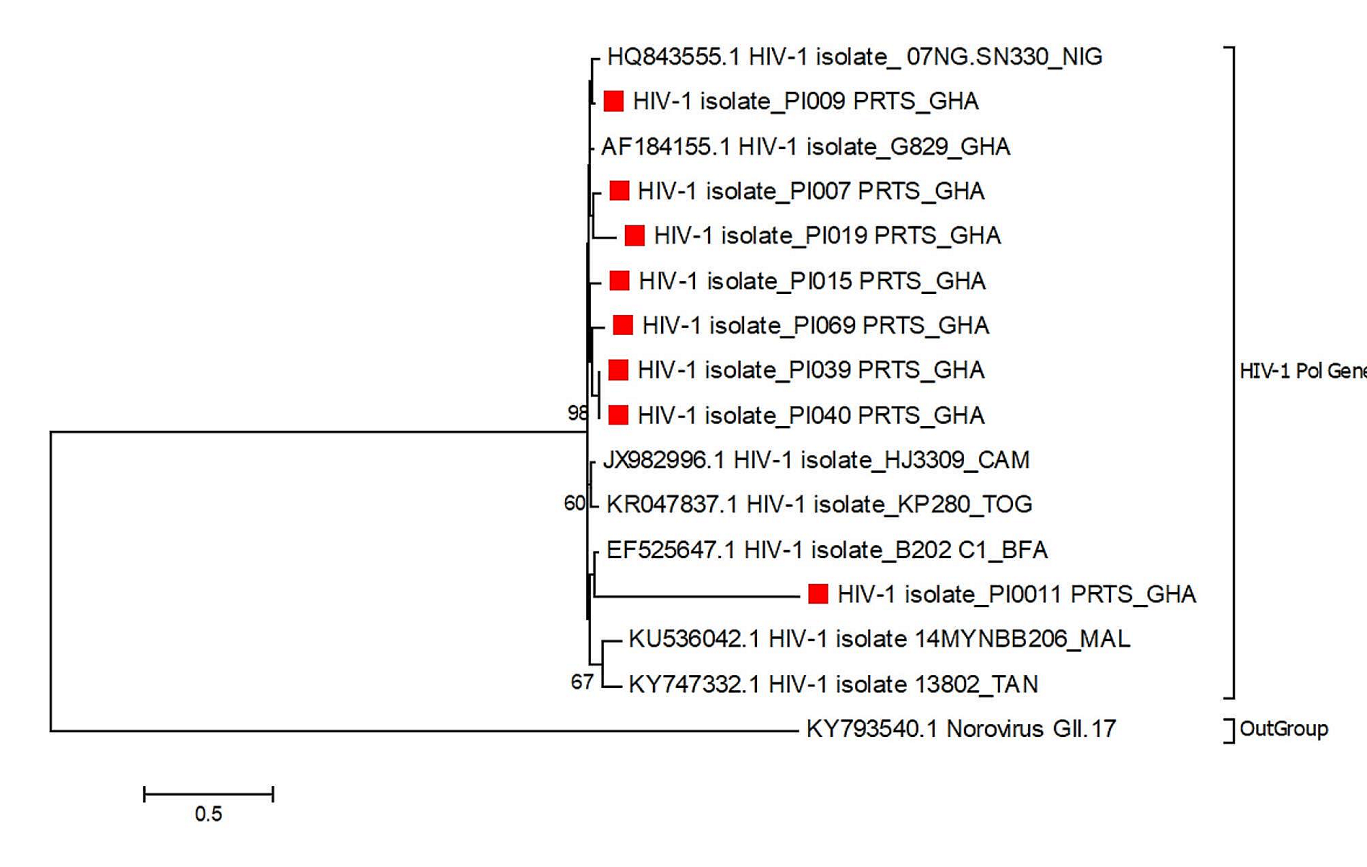
Phylogenetic analysis of *PR* gene sequences. A phylogenetic analysis using the eight successfully sequenced samples was done. Except for one sequence, PI0011_PRTS_GHA that clustered with the Burkinabe HIV-isolate B202 C1_BFA, study sequences clustered well with one Nigerian sequence (HQ843555.1 HIV-1 isolate_07NG.SN330_NIG). The red squares represent the sequences under consideration

## Discussion

This study sought to investigate the PI drug resistance mutations among PLWH virologically failing second line antiretroviral therapy. Most of the participants were females, and aged above 40 years, this agrees with other studies in Sub-Saharan Africa and the nationally reported incidence of HIV in Ghana where females happen to be more affected than males [2, 15].

In most Sub-Saharan African countries including Ghana, where there exist technical and financial challenges in routinely assessing viral load and drug resistance testing in patient care, assessing CD4^+^ T-cell counts and clinical symptoms are necessary to monitoring the success of ART, hence their continuous and consistent investigation is important [16]. In this study, a significant reduction in viral load was observed after 1 month of adherence but relatively stable CD4^+^ T cell count. The importance of monitoring viral load for patient management has been well communicated by the World Health Organisation (WHO) [17, 18]. In the recent 2023 International AIDS Society (IAS) conference in Australia, the WHO reported evidence of achieving better treatment outcomes and programmatic success such as in U = U (Undetectable = Untransmissible) when viral load is below < 1000 copies/ml [19]. Even though, monitoring CD4 + T cell count is accepted, it is not recommended and done occasionally when countries can afford the cost of routine viral load. Thus, most developed settings do not routinely use it in patient care as several clinical factors can affect the results [20].

Overall, the trends suggested that the second-line drugs used over the period had been effective, and only when used appropriately. Notwithstanding their effectiveness, only about a limited number of HIV patients in Ghana are on PI partly due to financial constraints and unavailability [10]. These findings confirmed the self-reported adherent behaviour from patients reporting low adherence rate of 97% for one month. This agrees with other studies that showed that patients with 100% adherence had reduced risk of developing resistance to PI boosted regimens [21]. Again, tertiary educational level significantly correlated with reduced viral load copies. Even though this would need a more prospective study to arrive at concrete conclusions, patients who fairly appreciate their clinical situation and the importance of taking their medication and assessing their viral load can benefit the patient and healthcare providers [22, 23].

The 463 bp fragments of the protease coding genes of the *pol* gene of HIV-1 were amplified and sequenced. All mutations were non-polymorphic and have been associated with LPV/r and ATV/r. It is worth noting that a significant number of PLWH in Ghana are on LPV/r and ATZ/r regimens [10]. Positions 46, 54 and 82 of the protease gene are the main sites for developing a high-level resistance to LPV/r [24], whereas positions 84 and 90 are known to cause high-level resistance to ATV/r [25]. The failure to continuously assess patients for drug resistance variants impact treatment success. The findings corroborates with studies in South Africa and Tanzania where a third of South African patients had PI-drug resistance mutations impacting treatment with darunavir-containing regimen [26] and those in Tanzania with treatment failure after 6–12 months of PI-containing therapy [27]. We recommend here the use a drug safety monitory protocol among clinicians as part of treatment guidelines. Even though the study measured viral load at 1 week and 1 month timepoints and studied fewer patients who consented to make their clinical folders accessible, the clinical picture of the patients in the study emphasizes the need for political and financial willingness to implement HIV viral load monitoring, and couple it with genotypic antiretroviral testing for PLWH failing therapy as a standard of care in Ghana, especially in the era of DTG roll-out.

## Conclusion

The results indicate that majority of patients who have previously failed first line therapy and currently receiving PI-based second-line ART at the Korle-Bu Teaching Hospital were still not virologically unsuppressed due to reported cases of non-adherence. This presents with programmatic concerns and thus, routine HIV-1 viral load and drug resistance testing is necessary to early detect virologic failure and guide treatment.

## Data Availability

The data sets analysed during this study are available with the corresponding author on request.
